# Machine learning models predict overall survival and progression free survival of non-surgical esophageal cancer patients with chemoradiotherapy based on CT image radiomics signatures

**DOI:** 10.1186/s13014-022-02186-0

**Published:** 2022-12-27

**Authors:** Yongbin Cui, Zhengjiang Li, Mingyue Xiang, Dali Han, Yong Yin, Changsheng Ma

**Affiliations:** 1 Department of Graduate, Shandong First Medical University and Shandong Academy of Medical Sciences, Jinan, China; 2 Department of Radiation Oncology, Shandong Cancer Hospital and Institute, Shandong First Medical University and Shandong Academy of Medical Sciences, Jinan, China; 3 Department of VIP Medical Services and Radiation Oncology, National Cancer Center/National Clinical Research Center for Cancer/Cancer Hospital, Chinese Academy of Medical Sciences and Peking Union Medical College, Beijing, China

**Keywords:** Esophageal squamous cell carcinoma, Radiomics, Progression free survival, Overall survival, Machine learning

## Abstract

**Purpose:**

To construct machine learning models for predicting progression free survival (PFS) and overall survival (OS) with esophageal squamous cell carcinoma (ESCC) patients.

**Methods:**

204 ESCC patients were randomly divided into training cohort (n = 143) and test cohort (n = 61) according to the ratio of 7:3. Two radiomics models were constructed by radiomics features, which were selected by LASSO Cox model to predict PFS and OS, respectively. Clinical features were selected by univariate and multivariate Cox proportional hazards model (*p* < 0.05). Combined radiomics and clinical model was developed by selected clinical and radiomics features. The receiver operating characteristic curve, Kaplan Meier curve and nomogram were used to display the capability of constructed models.

**Results:**

There were 944 radiomics features extracted based on volume of interest in CT images. There were six radiomics features and seven clinical features for PFS prediction and three radiomics features and three clinical features for OS prediction; The radiomics models showed general performance in training cohort and test cohort for prediction for prediction PFS (AUC, 0.664, 0.676. C-index, 0.65, 0.64) and OS (AUC, 0.634, 0.646.C-index, 0.64, 0.65). The combined models displayed high performance in training cohort and test cohort for prediction PFS (AUC, 0.856, 0.833. C-index, 0.81, 0.79) and OS (AUC, 0.742, 0.768. C-index, 0.72, 0.71).

**Conclusion:**

We developed combined radiomics and clinical machine learning models with better performance than radiomics or clinical alone, which were used to accurate predict 3 years PFS and OS of non-surgical ESCC patients. The prediction results could provide a reference for clinical decision.

**Supplementary Information:**

The online version contains supplementary material available at 10.1186/s13014-022-02186-0.

## Introduction

Esophageal cancer (EC) is the seventh incidence and sixth mortality malignant tumors in the world, Easten Asia shows the highest regional incidence rates in the worldwid^e^ [1]. Especially in China, which is the main cause of the heavy burden for Easten Asia [1].The main subtype of EC in China is esophageal squamous cell carcinoma (ESCC), which has a proclivity for earlier lymphatic spread, and is associated with a poorer prognosis [2]. Due to most ESCC patients are diagnosed at an advanced stage, the 5 years overall survival rate is less than 20% [3]. Furthermore, most patients with advanced or medial stage ESCC always lost the operation opportunity [4, 5].Therefore, chemoradiotherapy (CRT) or neoadjuvant chemoradiotherapy (NCRT) are effectively strategies to treat with ESCC [6]. Even though there are different treatment strategies, the recurrence and metastasis are still the main factors that affects the prognosis and patient’s survival [7]. Currently, the methods to predict prognosis of ESCC patients are mostly based on clinic risk factors, pathology and image, such as the patients characteristics like age, gender, treatment response, the tumor characteristics like location, size, differential, TNM stage et al., the pathology characteristics like lymphovascular invasion, the hematology test results like leukocyte, platelet [8–12]^.^ Nevertheless, the prediction of treatment outcomes based on images or clinic risk factors alone is too simply to represent the actually therapeutic effects.

Radiomics is considered as one of the most vital technical to predict the efficacy of ESCC treatment. At present, the radiomics extracted signatures are mostly based on computer tomography (CT) imaging, magnetic resonance imaging (MRI) or18F-flurodeoxyglucose positron emission tomography (18F-FDG PET) /CT imaging [13]. Nakajo et al [14] found texture features intensity variability (IV) and size-zone variability (SZV), and volumetric parameters metabolic tumor volume (MTV) and total lesion glycolysis (TLG) can predict tumor response. And the positive and negative predictive values for non-responders were 77% and 69% in MTV, 76%and 100% in TLG, 78% and 67% in IV and78% and82% in SZV, respectively. But none of them was an independent factor for the prediction of prognosis in the EC patients. Furthermore, Li et al [15] recruited 134 ESCC treated by CRT patients to evaluate the prognostic value of metabolic parameters of pre-treatment and interim 18F-FDG PET/CT for overall survival (OS). And they found that maximum of standard uptake value (SUVmax2), MTV1, △MTV, TLG1, TLG2 and △TLG were associated with OS. However, the model with more robustness is urgently needed develop to predict the prognosis and OS of ESCC patients. Chu et al [16] developed an optimal model to predict disease-free survival (DFS) and overall survival (OS) in patients with ESCC and demonstrated MR image combined with clinical features had superior performance in both training and test groups for predicting DFS ([C-Index], 0.714, 0.729) and OS ([C-Index], 0.730, 0.712). Peng et al. [17] combined CT radiomics features and clinical risk factors to determine recurrence-free survival (RFS) and OS after surgery in patients with ESCC. They revealed that the C-indices of the RFS radiomics nomograms were 0.758, 0.722 and 0.684, and the C-indices of the OS radiomics nomograms were 0.884, 0.809 and 0.729 in the training cohort, internal validation cohort and external validation cohort, respectively.

This study is aimed to develop and test the radiomics models to predict 3 years progression free survival (PFS) and OS of no-surgical ESCC patients based on contrast enhanced CT(CECT) images, which combined radiomics features and clinical features. The models could be used in individualized evaluation in pre-treatment and providing decision-making reference.

## Materials and methods

### Patients

Our study recruited ESSC patients from February 2012 and December 2018, who were treated by chemoradiotherapy (CRT) in Shan Dong first medical university affiliated tumor hospital. Inclusion criteria were as follows: (1) age ≥ 18; (2) Eastern Cooperative Oncology Group performance status (ECOG PS) ≤ 2; (3) histopathologically confirmed squamous cell carcinoma; (4) cT3-4N0M0/cT1-4N + M0 or cM1 (positive nonregional lymph nodes and irradiated during radiotherapy) in accordance with AJCC 7th edition; (5) treated by 3-dimensional conformal radiation therapy(3D-CRT) or intensity-modulated radiation therapy (IMRT) with radiation total doses ≥ 50 Gy using conventional fractionated radiotherapy, chemotherapy cycles ≥ 4, chemotherapy with cisplatin plus fluorouracil (PF) or docetaxel (DP). Exclusion criteria were as follows: (1) patients changed chemotherapy regimens during definitive chemoradiotherapy; (2) CT images quality were poorly; (3) patients who underwent radical surgical treatment. This study was approved by the ethics committee of Shandong First Medical University affiliated tumor hospital according to the Declaration of Helsinki. Due to the research was a retrospective scientific study, there was no informed consent form in our investigation. All the patients were randomly divided into training cohort and test cohort according to the proportion of 7:3.

### The acquisition of CT images and the region of interest

All the ESCC patients were scanned with Philips Big Core CT (Phillips Medical Systems, 96 Highland Heights, OH). The scanning parameters were as follows: tube voltage: 120KvP, tube current: 53-400 mA, each scanning period: 2.8 s, the interval time: 1.8 s. The CT images were reconstructed with a 512 × 512 image matrix and a voxel size of 0.9766 mm × 0.9766 mm × 3 mm. The image thickness was 3 mm. Patients were fixed by a vacuum cushion in the scanning process. Afterwards, the intravenous CECT images of each patient for the development of treatment planning.

The volume of interest (VOI) was defined as the gross tumor volume (GTV), which was the visible primary tumor (GTVp) and metastatic lymph nodes (GTVnd) detected by CECT. A radiologist with more than 10-year work experience delineated the VOI according to the National Comprehensive Cancer Network (NCCN) guideline. And another senior radiologist reviewed the delineation.

### The collection of clinical features

The clinical features were collected, which involved: age, gender, tumor location, TNM stage, differentiation, therapeutic model, radiotherapy technology and dose, chemotherapy plan and cycles, the hematology test results, radiation pneumonia (RP), radiation esophagitis (RE), nausea or vomiting (NV), cardiac disorders, clinical response, Objective Response Rate (ORR), Disease Control Rate (DCR). Patients were followed up every 1 to 3 months after completion of chemotherapy for the first 2 years and every 6 to 12 months thereafter.

Tumor’s location and clinical TNM stage was evaluated by the medical imaging examination, such as CT, PET-CT. All the patients were treated with four different CRT therapeutic models, which included induction chemotherapy followed by concurrent chemoradiotherapy (I-CCRT), concurrent chemoradiotherapy followed by consolidation chemotherapy (CCRT-C), induction chemotherapy followed by concurrent chemoradiotherapy and consolidation chemotherapy (I-CCRT-C), sequential chemoradiation (SCRT). The ECOG PS of ESCC patients were assessed by the patient’s performance. The differentiation of cancer cell was estimated by the pathological examination. Some hematology test values were involved in our research, such as carcinoembryonic antigen (CEA), Cytokeratin-19-fragment CYFRA21-1 (Cyfra21). Others hematology test results were also classified to different grades by the Common Terminology Criteria for Adverse Events Version 4.0 (CTCAE 4.0), which included anemia, leukopenia, thrombocytopenia, neutropenia, aspartate aminotransferase (AST), alanine transaminase (ALT), total bilirubin (TBIL). What’s more, the RE, RP, NV and Cardiac disorders were classified or showed in the study. Such as ORR, DCR, response, the efficacy was evaluated by the Response Evaluation Criteria in Solid Tumors Version 1.0. And clinical response was classified as complete response (CR), partial response (PR), no response (NR), or progressive disease (PD). PFS was defined as the period from the start of the anticancer treatment to the time of the first diagnostic progression or death or last follow-up. OS was defined from the start of the initial antitumor treatment to the date of death from any cause, regardless of disease status or last follow-up.

### Feature extraction

We extracted the radiomics features from the basal CT before any therapy and the clinical parameters collected from the first follow-up CTs. Patients were followed up every 1 to 3 months after completion of chemotherapy for the first 2 years and every 6 to 12 months thereafter. Total of 944 features based on patient CT images were extracted by Radiomics software based on 3D slicer, which were divided into two categories: without preprocessing and after wavelet transform. In addition, these features include 14 shape features, 180 first-order features and 750 texture features. The resampled voxel sizes were set to 3 × 3 × 3 mm^3^ to standardize the slice thickness. The radiomics features were generated from the original, wavelet-filtered, and Laplacian of Gaussian (LoG)-filtered images. And the Log-kernel size was set to 3 × 3. The features included shape, shape2D, First-order and texture feature. Texture features included Gray Level Cooccurrence Matrix (GLCM), Gray Level Dependence Matrix (GLDM), Gray Level Run Length Matrix (GLRLM), Gray Level Size Zone Matrix (GLSZM) and Neighborhood Gray-tone Difference Matrix (NGTDM) [18].

### Feature selection and model development

A total of 204 ESCC patients were divided into training and test cohorts to evaluate 3 years PFS and OS. Based on the training cohort, radiomics features were selected by the least absolute shrinkage and selection operator (LASSO) Cox model with tenfold cross validation, respectively. According to the selected radiomics features, two radiomics models with good prediction performance for PFS and OS were established.

Clinical features were selected by univariate and multivariate Cox proportional hazards model (*p* < 0.05). The selected clinical features were added into the multivariable Cox proportional hazards model based on radiomics features to improve the predictive ability. Finally, two combined models were established by selected radiomics and clinical features to predict PFS and OS.

**Fig. 1:**
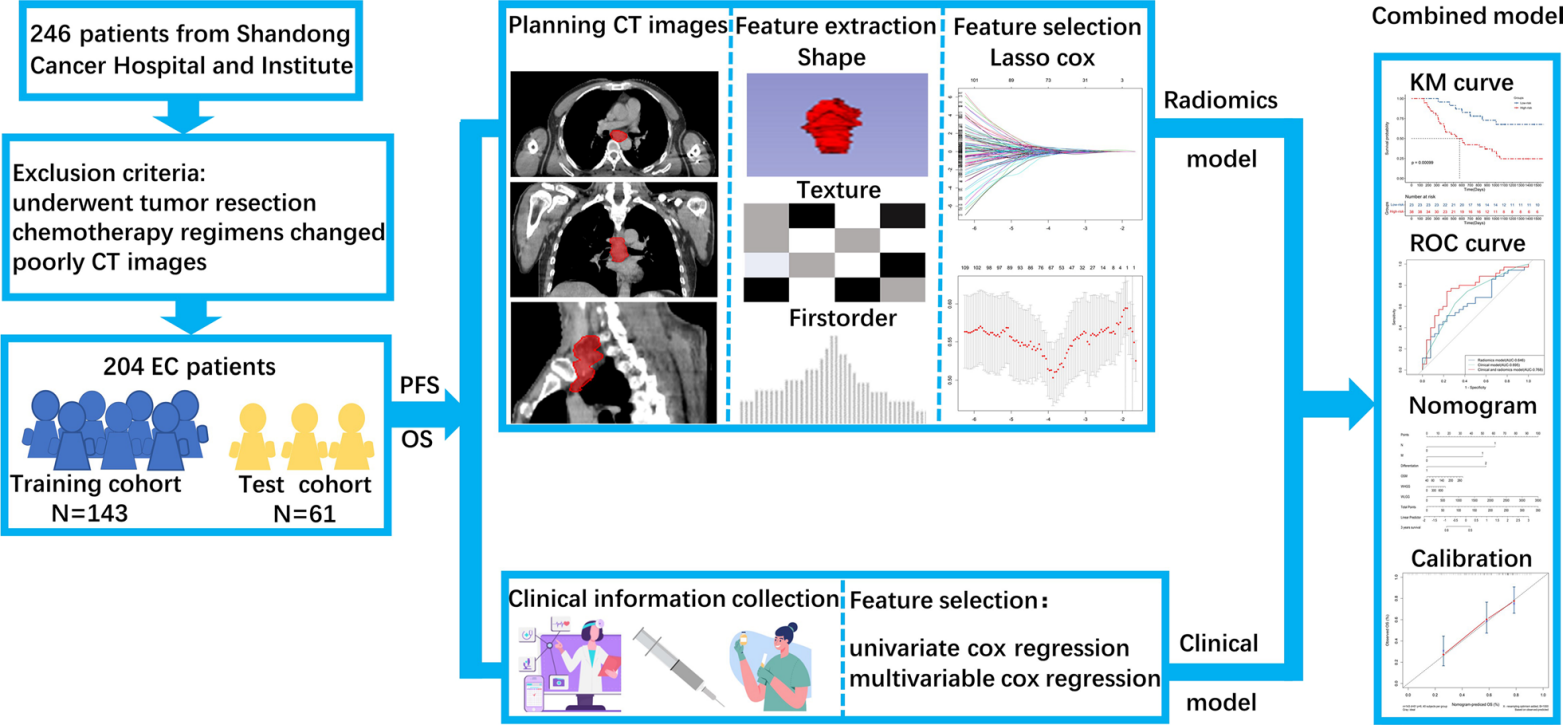
The flow chart of this study. PFS, progression free survival; OS, overall survival; KM, Kaplan–Meier; ROC, receiver operator characteristic

The optimum cutoff value was the median of predicted value. Consequently, patients were divided into a high-risk group and a low-risk group in the training set. After the survival curves of the two groups were evaluated by the Kaplan Meier (KM) method, the differences between the survival curves were tested by the log-rank test (*p* < 0.05). The prediction ability of the survival rate was evaluated by the concordance index (C-index) and receiver operator characteristic (ROC) curve. Nomograms and calibration curve were built based on the two clinical and radiomics models. Calibration curves were calculated to evaluate the consistency between the nomogram-predicted results and recorded survival results. The flowchart of survival model construction is presented in Fig. 1.

### Statistical analysis

Feature extraction was implemented in 3DSlicer (Version 4.11, https://www.slicer.org/). Statistical analyses were performed using R software (Version 3.4.0, https://www.r-project.org/). The Kruskal–Wall test performed in MATLAB (2013b, https://www.mathworks.com/) was used to analyze the different groups, and *P* values less than 0.05 were considered statistically significant. All statistical tests were two-sided.

## Results

### Patient demographics and clinicopathological characteristics

A total of 204 patients were analyzed to predict 3 years PFS and OS, respectively. The clinicopathological characteristics for survival analysis (training and test cohort) are shown in Additional file 5: Table S1. There were no significant differences between training cohort and test cohort of the clinical variables, except ECOG PS and Differentiation in PFS prediction model and response, DCR, ORR in OS prediction model. All of the advanced non-surgical ESCC patients were followed up for the full length of the follow-up period. In end of the last follow-up, 87 patients (60.84%) in the training set and 38 patients (62.30%) in the test set had disease progression. The survival time of 61 patients (42.66%) in the training set and 35 patients (57.38%) in the test was less than three years. There was no statistically significant difference in PFS and OS between the two groups (χ^2^ = 0.038, *P* = 0.845; χ^2^ = 3.719, *P* = 0.054, respectively).

### The establishment of models to predict PFS

There were 944 radiomics features extracted from the VOI of CT images. Six radiomics features were selected by LASSO Cox model (Additional file 1: Figure S1a, 1b). Radiomics model was built by these features. Univariate and multivariate Cox hazard regression models were used to select clinical features, including ECOG PS, N stage, differentiation, RE, ORR, DCR, clinical response, (*p* < 0.05, respectively). The analysis of all clinical features was shown in Table S2. The radiomics features were shown in Additional file 7: Table S3. Combined model was built by selected radiomics and clinical features.

**Fig. 2:**
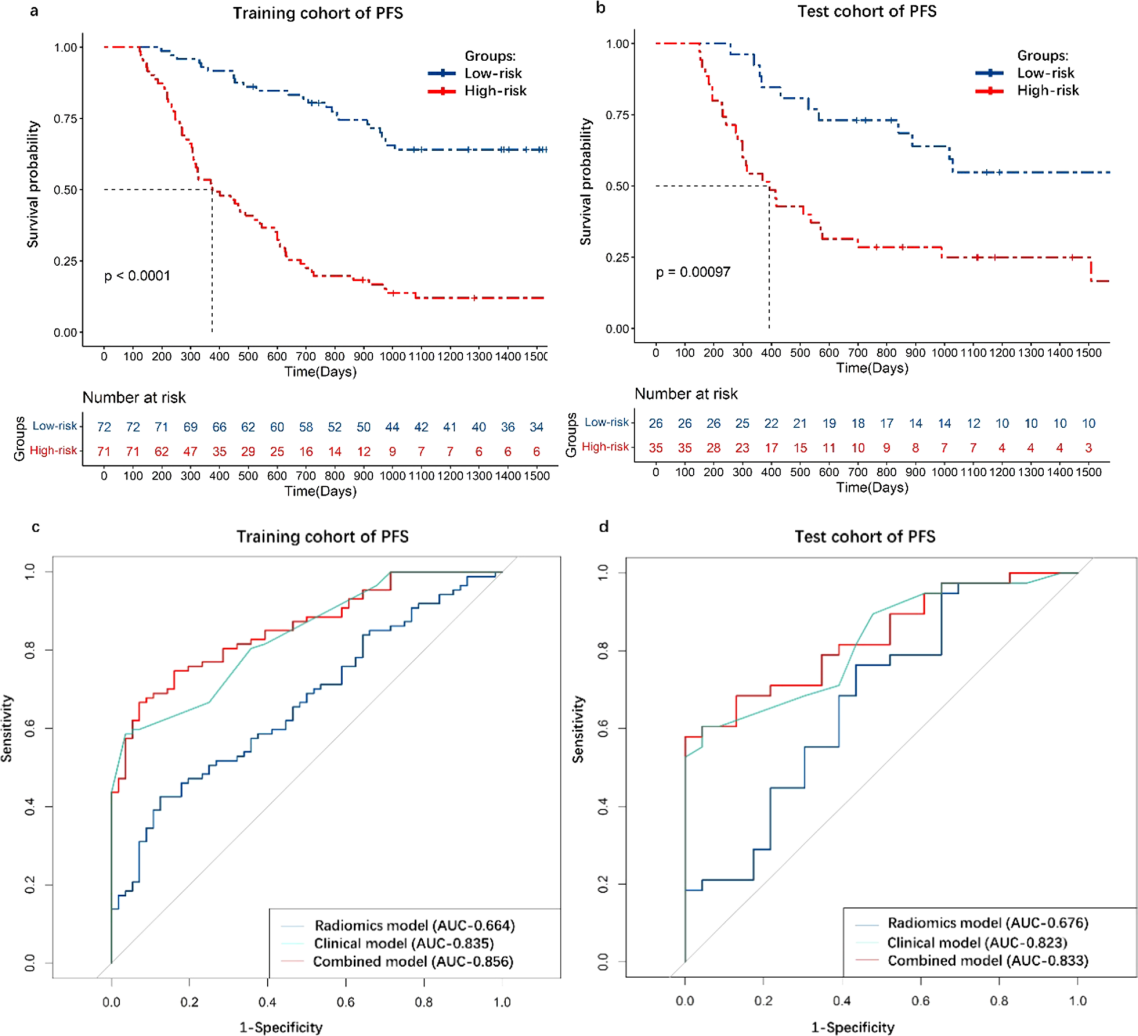
The KM curve and ROC curve of PFS prediction model. **a** KM curve in training cohort. **b** KM curve in test cohort. **c** ROC curve in training cohort. **d** ROC curve in test cohort. PFS, progression free survival; OS, overall survival; KM, Kaplan–Meier; ROC, receiver operator characteristic

As the KM curves shown (Fig. 2a, b), selected radiomics and clinical features discriminated between high-risk group and low-risk group. In the training cohort, the C-index of radiomics model, clinical model, combined model was 0.65, 0.79 and 0.81, the AUC was 0.664, 0.835 and 0.856 respectively (Fig. 2c). In the test cohort, the C-index of radiomics model, clinical model, combined model was 0.64, 0.78 and 0.79, the AUC was 0.676, 0.823 and 0.833, respectively (Fig. 2d). The nomogram was constructed by combined model (Additional file 2: Fig. S2a). Then, the calibration curves of the nomogram for PFS showed that the predicted value of 3 years of PFS was roughly consistent with the actual value (Additional file 2: Fig. S2b, 2c).

### The establishment of models to predict OS

Radiomics model was built by three radiomics features which were selected by LASSO Cox model (Additional file 3: Figure S3a, 3b). Univariate and multivariate Cox hazard regression models were used to choose clinical features, including N stage, M stage and differentiation (*p* < 0.05, respectively). The analysis of all clinical features was shown in Additional file 5: Table S2. The radiomics features were shown in Additional file 8: Table S4. Combined model was built by selected radiomics and clinical features.

**Fig. 3:**
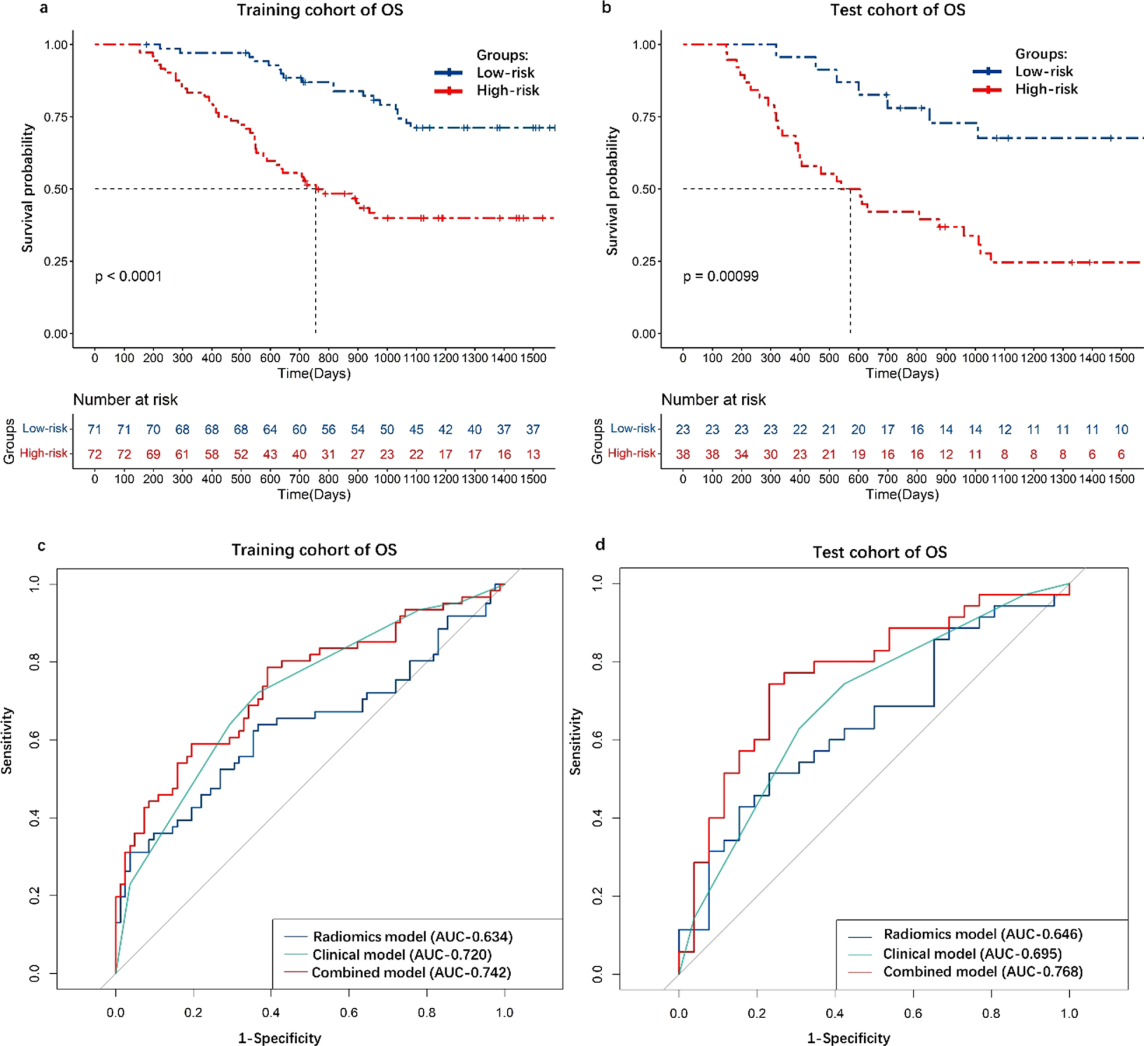
The KM curve and ROC curve of PFS prediction model. **a** KM curve in training cohort. **b** KM curve in test cohort. **c** ROC curve in training cohort. **d** ROC curve in test cohort. PFS, progression free survival; OS, overall survival; KM, Kaplan–Meier; ROC, receiver operator characteristic

As the KM curves shown (Fig. 3a, b), selected radiomics and clinical features discriminated between high-risk group and low-risk group. In the training cohort, the C-index of radiomics model, clinical model, combined model was 0.64, 0.69, 0.72, respectively and the AUC was 0.634, 0.720, 0.742, respectively (Fig. 3c). In the test cohort, the C-index of radiomics model, clinical model, combined model was 0.65, 0.64 and 0.71, respectively. The AUC was 0.646, 0.695 and 0.768, respectively (Fig. 3d). The nomogram was constructed by combined model (Additional file 4: Fig. S4a). Then, the calibration curves of the nomogram for OS showed that the predicted value of 3 years of OS was roughly consistent with the actual value (Additional file 4: Fig. S4b, 4c).

## Discussion

Radiomics studies in EC most focused on the prediction of lymph node metastasis, radiation-induced diseases in the earlier [19–21]. What’s more, there were still few researches in the prediction of both PFS and OS [16, 22–24]. The establishment of accurate prediction models of PFS and OS were conducive to clinical decision-making and are expected to improve the survival rate of advanced ESCC patients. Therefore, in the present study, we constructed and validated machine learning models to predict PFS and OS of non-surgical ESCC patients, which incorporated the clinical variables and CECT images. The C-index and AUC showed that combined models had a better performance than radiomics or clinic models alone. The results demonstrated that incorporated the clinical variables enhanced the combined models’ predictive efficacy both in PFS and OS. In other word, the pre-treatment CECT images would not provide enough information to predict the treatment outcomes and the clinical data are essential for patient’s survival prediction.

As for the prediction of PFS and OS, the combined models performed well with the prognostic accuracy over 70% based on clinical and radiomics features. The C-index of PFS prediction radiomics model, clinical model, combined model in the test cohort was 0.64, 0.78, 0.79 and AUC was 0.676, 0.823, 0.833, respectively. The C-index of OS prediction radiomics model, clinical model, combined model in the test cohort was 0.65, 0.64, 0.71 and the AUC was 0.646, 0.695, 0.768, respectively. Bohanes et al. [25] found that gender and age had significant influence for the treatment outcomes of EC patients. But, in our study, age and sex were not involved in the model’s development as they were no statistically significant by univariate Cox regression. TNM stage was the most commonly prognosis prediction method in clinical. MES et al. [26] and Zhao et al. [27] combined TNM stage and other clinical factors to improve the prognostic predictive. In the PFS and OS prediction, N stage was filtered to develop clinical model in our study. But for the prediction of OS, M stage was also selected to develop models. Li et al. [28] used deep learning to predict the treatment response to CCRT for ESCC patients which also included the M stage in the progress of model development.

Jayaprakasam et al. [22] established radiomics model to predict PFS based on 72 ESCC patient’s PET/CT images and the AUC was 0.73 in the test cohort. They first included the PET responders into survey. But, PET/CT examination was highly expensive than CT or MRI and the sample was small in the study. Luo et al. [23] also developed a nomogram model for predicting local PFS based on CT images and C-index was 0.723 in test cohort. This study included clinical response to develop model and obtained a fine result. Liu et al. [29] have found that clinical complete response after neoadjuvant CRT was significantly correlated with survival of patients with ESCC. So, we also selected the clinical response, ORR and DCR into model’s building, which significantly enhanced the model’s prediction efficacy of PFS. After selecting, the tumor differentiation was also chosen to develop the PFS and OS prediction models. Barbetta et al. [30] found that poor tumor differentiation was an independent risk factor for recurrence in EC patients. Qiu et al. [31]incorporated radiomics and clinical features (including tumor differentiation) to predict postoperative recurrence risk of ESCC patients.And the C-index of test cohort was 0.724 in their combined model. In previous study, researchers had proved that ECOG PS had significant prognostic effects on clinical response and survival [32, 33].And RE was regarded as one of the factors affecting patient’s prognosis [34]. ECOG PS was used to evaluated the patient’s physical performance before treatment and RE was the radiation-induced esophageal disease after treatment. In our study, ECOG PS and RE were also selected to predict PFS.

Except the tumor differentiation and N stage described above, M stage was also selected to develop the OS prediction models. Shi et al. [35] concluded that metastatic lesions were closely related to the prognosis of patients. Li et al. [28] used deep learning to predict the treatment response to CCRT for ESCC patients and the M stage was also involved in the progression of model development. Due to the heterogeneity of tumor, the treatment outcomes of patients might be different even with the same clinical features [11, 36, 37].

Radiomics is defined as the high-throughput extraction of image features from radiographic images [38, 39]. Radiomic features provide abundant additional information predictive of underlying tumor biology and behavior [40]. These signatures can be used alone or with other patient related data (e.g., pathological data, genomic data, clinical data) to predict tumor phenotyping, treatment response prediction and prognosis. Our study finial selected one shape texture, two GLSZM textures, two GLDM textures and one GLCM texture to develop PFS prediction model and one shape texture, one GLSZM texture, one GLDM texture to construct OS prediction model. Wavelet transformed features contain more information and are more difficult to explain than first-order and shape features, but also reflect more complex information about tumor heterogeneity [16]. Therefore, the prediction results based on Wavelet transformed features are consistent with the cognition of clinical outcomes.

Although our research developed the survival prediction model with good accuracy, there are still some limitations in our study. Due to it is a retrospective study, some clinical variables are not comprehensive enough. Furthermore, all of the recruited EC patients were confirmed ESCC by pathology examination, which were the advanced stage and lost the surgery opportunity. Therefore, the constructed model may be limited in esophageal adenocarcinoma (EAC) or surgery patients. Due to the treatment plan is so vital for patient’s survival, our study recruited the patients treated with CRT, which might limit the model’s therapy decision. Adding the genomics features in the model is willing to improve the accuracy of the treatment outcomes. What’s more, the further study of Delta-radiomics model could provide better prediction for the PFS and OS [41].

## Conclusion

This research displays a great performance for the prediction ESCC patients PFS and OS by combining the pre-treatment CECT radiomics signatures with clinical features. The prediction results will provide further decision-marking reference for clinician.

## Supplementary Information

**Additional file 1: Fig. S1.** Radiomics feature s selected by LASSO Cox for PFS prediction.

**Additional file 2: Fig. S2.** PFS prediction Nomogram and calibration curve of combined model.

**Additional file 3: Fig. S3.** Radiomics features selected by LASSO Cox for OS prediction.

**Additional file 4: Fig. S4.** OS prediction Nomogram and calibration curves of combined model.

**Additional file 5: Table S1.** Patient characteristics in training (n = 143) and test cohorts (n = 61).

**Additional file 6: Table S2.** The clinical characteristics analysis of PFS and OS in the training cohort.

**Additional file 7: Table S3.** Radiomics features selected with LASSO Cox for PFS prediction.

**Additional file 8: Table S4.** Radiomics features selected with LASSO Cox for OS prediction.

## Data Availability

All data generated or analyzed during this study are available from the corresponding author on reasonable request.

## Abbreviations

EC: Esophageal cancer
ESCC: Esophageal squamous cell carcinoma
CRT: Chemoradiotherapy
NCRT: Neoadjuvant chemoradiotherapy
CT: Computer tomography
MRI: Magnetic resonance imaging
18F-FDG PET: 18F-flurodeoxyglucose positron emission tomography
OS: Overall survival
RFS: Recurrence-free survival
PFS: Progression free survival
CECT: Contrast enhanced CT
ECOG PS: Eastern Cooperative Oncology Group performance status
3D-CRT: 3-Dimensional conformal radiation therapy
IMRT: Intensity-modulated radiation therapy
PF: Plus fluorouracil
DP: Plus docetaxel
VOI: Volume of interest
GTV: Gross tumor volume
GTVp: Primary tumor
GTVnd: Metastatic lymph nodes
RP: Radiation pneumonia
RE: Radiation esophagitis
NV: Nausea or vomiting
ORR: Objective response rate
DCR: Disease control rate
I-CCRT: Induction chemotherapy followed by concurrent chemoradiotherapy
CCRT-C: Concurrent chemoradiotherapy followed by consolidation chemotherapy
I-CCRT-C: Induction chemotherapy followed by concurrent chemoradiotherapy and consolidation chemotherapy
SCRT: Sequential chemoradiotherapy
CEA: Carcinoembryonic antigen
AST: Aspartate aminotransferase
ALT: Alanine transaminase
TBIL: Total bilirubin
ULN: Upper limit of normal value
CR: Complete response
PR: Partial response
NR: No response
PR: Progressive disease
GLCM: Gray level cooccurrence matrix
GLDM: Gray level dependence matrix
GLRLM: Gray level run length matrix
GLSZM: Gray level size zone matrix
NGTDM: Neighborhood gray-tone difference matrix
LASSO: Least absolute shrinkage and selection operator
KM: Kaplan Meier
C-index: Concordance index
ROC: Receiver operator characteristic
ECA: Esophageal adenocarcinoma
