# Supplementary material for: Machine learning models predict overall survival and progression free survival of non-surgical esophageal cancer patients with chemoradiotherapy based on CT image radiomics signatures
Source: Radiat Oncol. 2022 Dec 27;17:212. doi: 10.1186/s13014-022-02186-0 (PMC9795769; doi:10.1186/s13014-022-02186-0)
Supplement: Supplementary file 2 — Additional file 2: Fig. S2. PFS prediction Nomogram and calibration curve of combined model. [file 13014_2022_2186_MOESM2_ESM.docx]

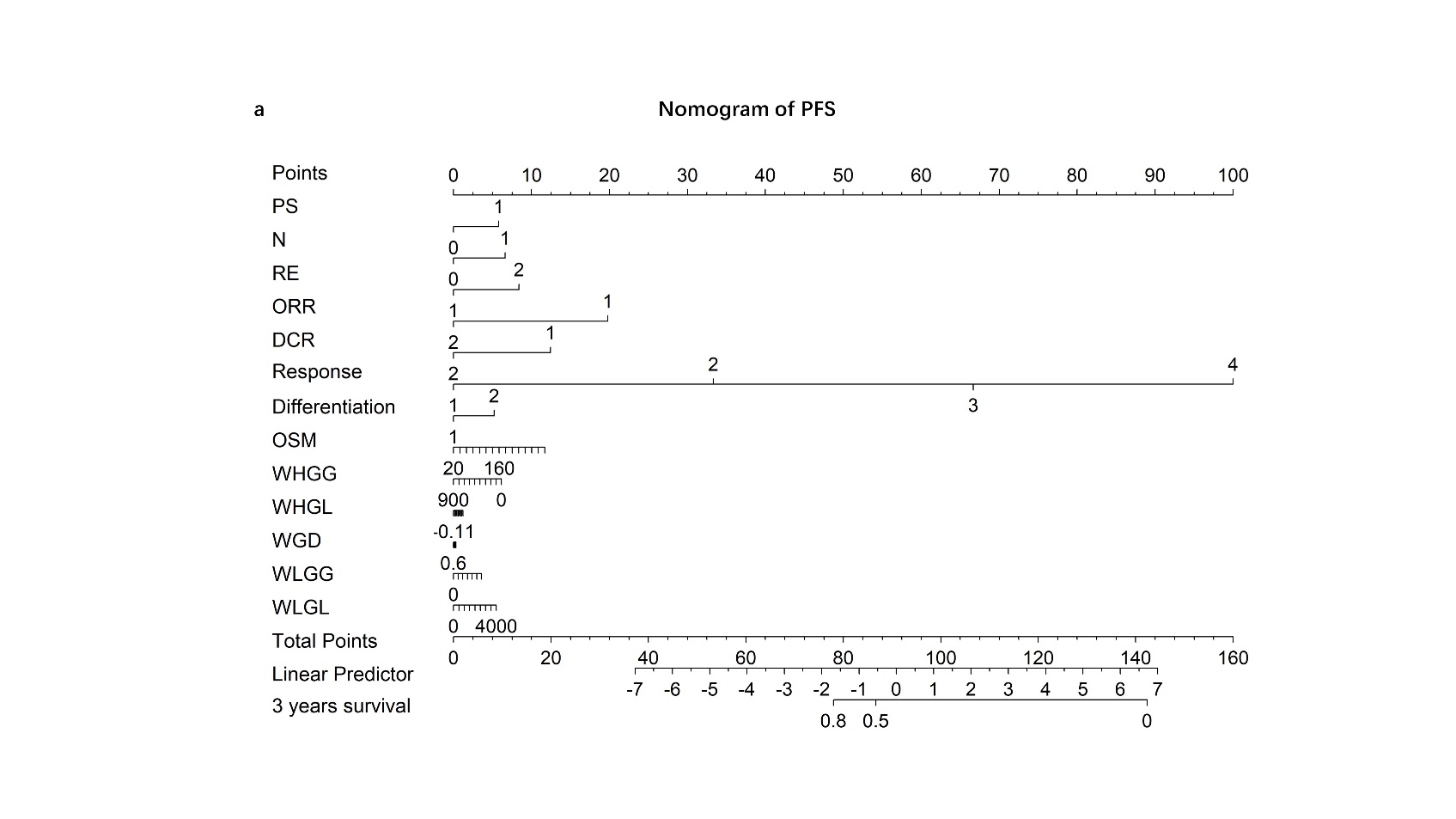


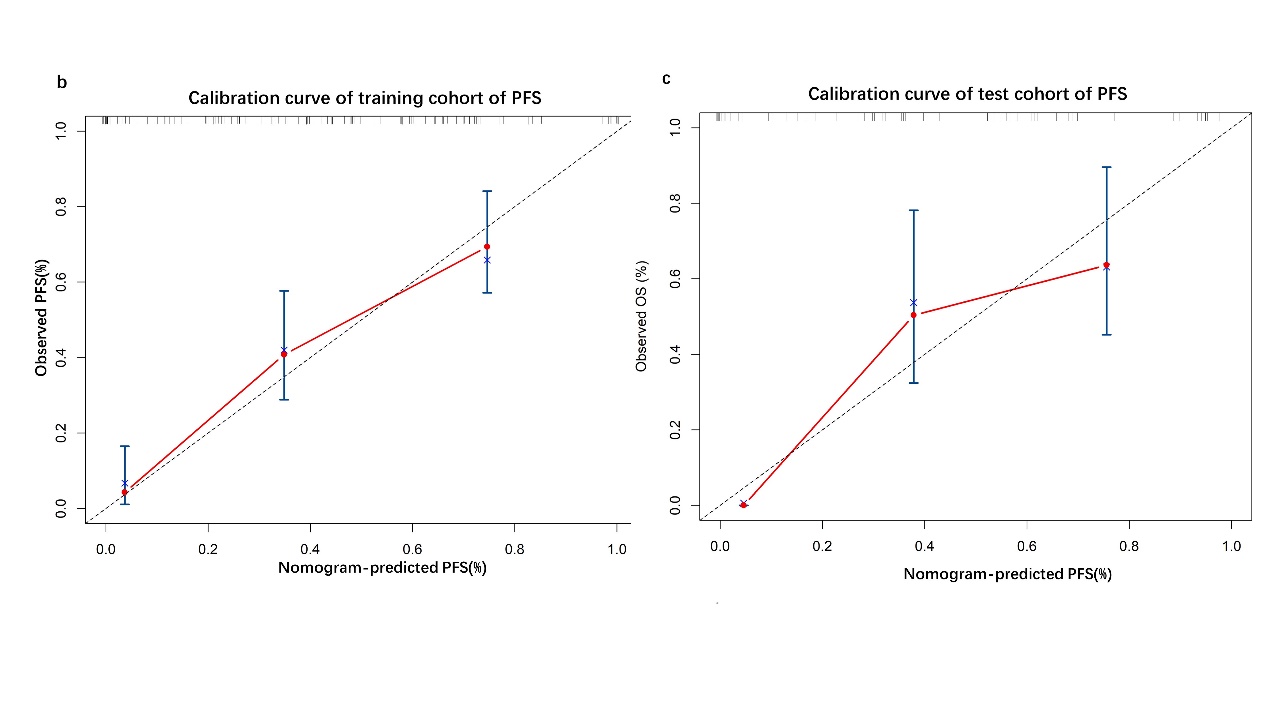


Figure S2 PFS prediction Nomogram and calibration curve of combined model. PFS: Progression

free survival PS: Performance status; N: node metastasis; RE: Radiation esophagitis; ORR: Objective response rate; DCR: Disease control rate; OSM: original, shape, Maximum2DDiameterRow; WHGG: wavelet-HLH, glszm, GrayLevelNonUniformity; WHGL: wavelet-HHL, glcm, Imc1; WGD: wavelet-HHH, gldm, DependenceNonUniformity; WLGG: wavelet-LLL，gldm，GrayLevelNonUniformity; WLGL: wavelet-LLL,glszm, LargeAreaEmphasis
