# Supplementary material for: Machine learning models predict overall survival and progression free survival of non-surgical esophageal cancer patients with chemoradiotherapy based on CT image radiomics signatures
Source: Radiat Oncol. 2022 Dec 27;17:212. doi: 10.1186/s13014-022-02186-0 (PMC9795769; doi:10.1186/s13014-022-02186-0)
Supplement: Supplementary file 3 — Additional file 3: Fig. S3. Radiomics features selected by LASSO Cox for OS prediction. [file 13014_2022_2186_MOESM3_ESM.docx]

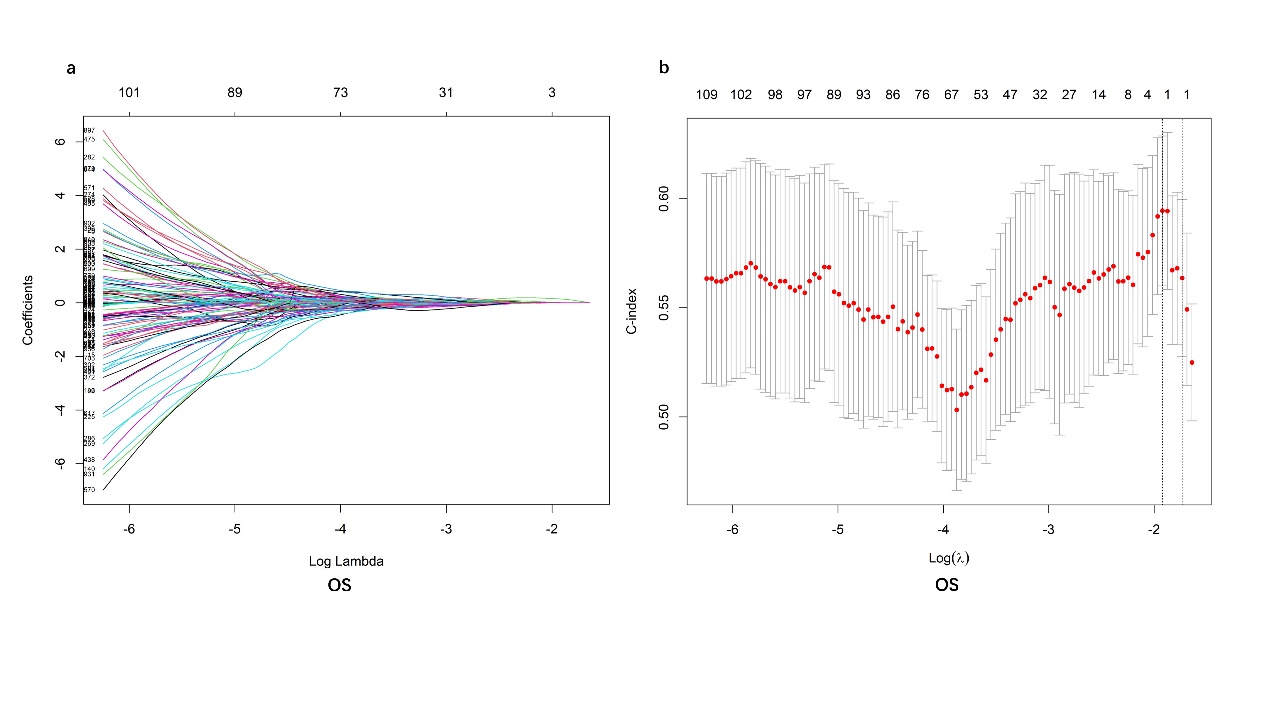


Figure S3 Radiomics features selected by LASSO Cox for OS prediction. OS: Overall survival; C-index: Concordance index;
