# Supplementary material for: Machine learning models predict overall survival and progression free survival of non-surgical esophageal cancer patients with chemoradiotherapy based on CT image radiomics signatures
Source: Radiat Oncol. 2022 Dec 27;17:212. doi: 10.1186/s13014-022-02186-0 (PMC9795769; doi:10.1186/s13014-022-02186-0)
Supplement: Supplementary file 4 — Additional file 4: Fig. S4. OS prediction Nomogram and calibration curves of combined model. [file 13014_2022_2186_MOESM4_ESM.docx]

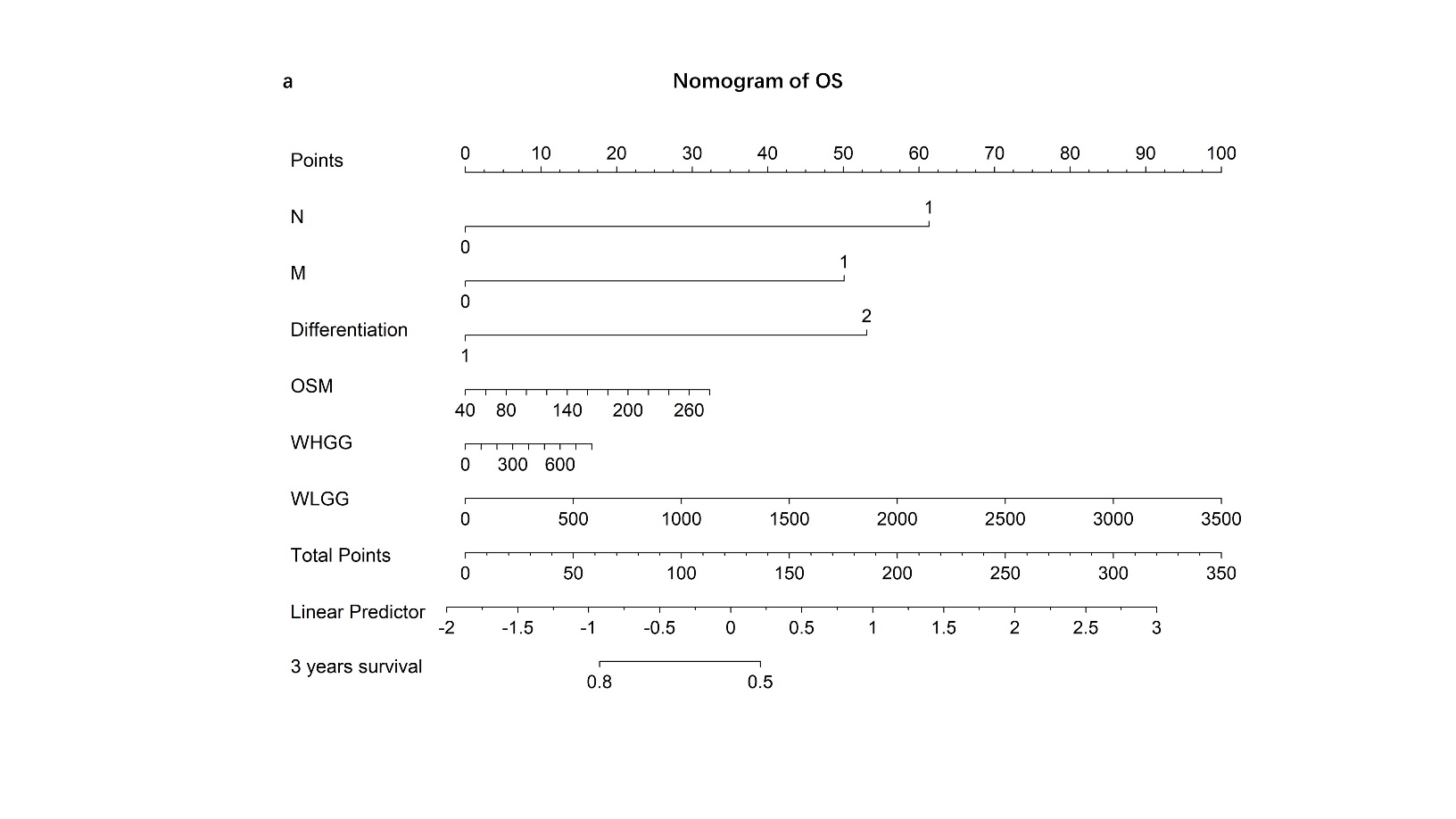


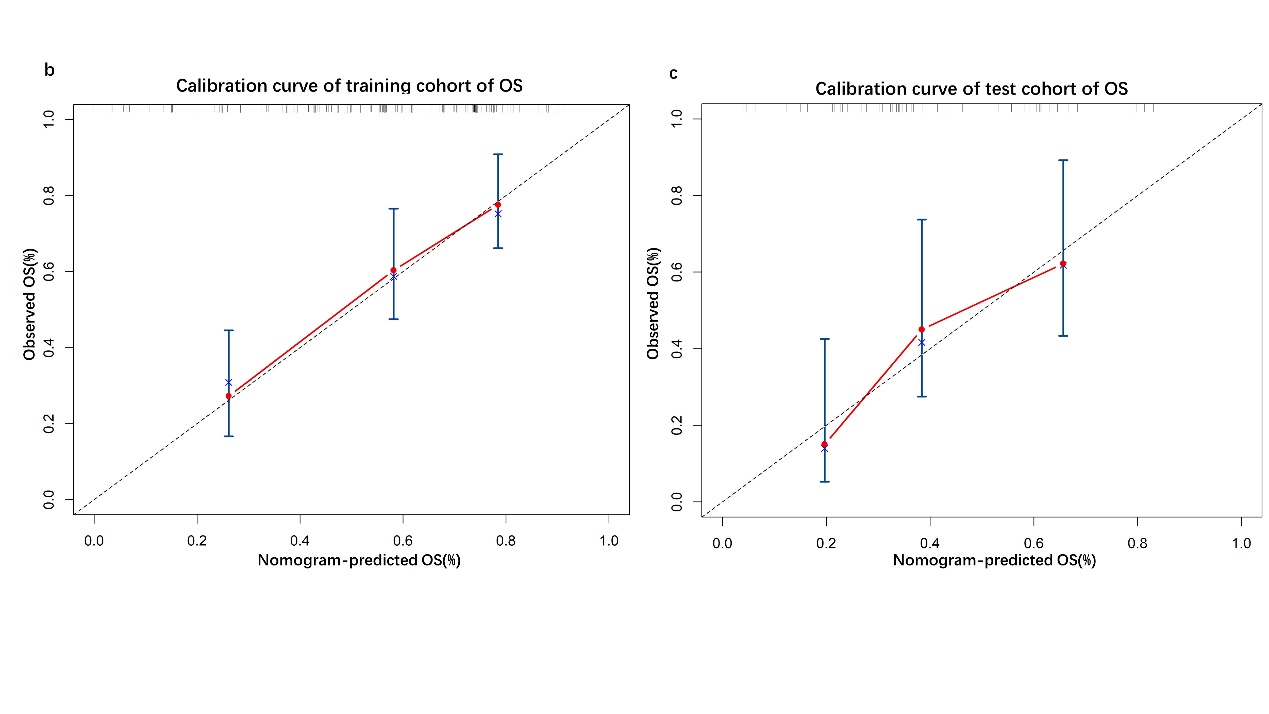


Figure S4. OS prediction Nomogram and calibration curves of combined model. Calibration curves for the nomogram in training cohort and test cohort. N: node metastasis; M: Metastasis; OSM: original, shape, Maximum2DDiameterRow; WHGG: wavelet-HLH, glszm, GrayLevelNonUniformity; WLGG: wavelet-LLL，gldm, GrayLevelNonUniformity;
