# Supplementary material for: Machine learning models predict overall survival and progression free survival of non-surgical esophageal cancer patients with chemoradiotherapy based on CT image radiomics signatures
Source: Radiat Oncol. 2022 Dec 27;17:212. doi: 10.1186/s13014-022-02186-0 (PMC9795769; doi:10.1186/s13014-022-02186-0)
Supplement: Supplementary file 5 — Additional file 5: Table S1. Patient characteristics in training (n = 143) and test cohorts (n = 61). [file 13014_2022_2186_MOESM5_ESM.docx]

Table S1. Patient characteristics in training (n=143) and test cohort (n=61).

|  | PFS | |  | OS | |  |
| --- | --- | --- | --- | --- | --- | --- |
|  | Training cohort | Test  Cohort | *P* values | Training cohort | Test  cohort | *P* values |
| **Clinical characteristic** | | | | | | |
| Age (years, median-range) | 62(44-79) | 62(39-78) | 0.568 | 63(39-79) | 59(44-74) | 0.065 |
| Gender (Male/Female) | 114/29 | 49/12 | 0.921 | 115/28 | 48/13 | 0.778 |
| ECOG PS |  |  | 0.020 |  |  | 0.904 |
| PS=1 | 77 | 22 |  | 69 | 30 |  |
| PS=2 | 66 | 39 |  | 74 | 31 |  |
| Tumor location |  | | 0.687 |  | | 0.792 |
| Cervical | 19 | 9 |  | 19 | 9 |  |
| Upper | 52 | 23 |  | 52 | 23 |  |
| Middle | 54 | 22 |  | 55 | 21 |  |
| Lower | 18 | 7 |  | 17 | 8 |  |
| Differentiation |  |  | 0.005 |  |  | 0.313 |
| Well | 78 | 20 |  | 72 | 26 |  |
| Poorly /middle | 65 | 41 |  | 71 | 35 |  |
| T stage |  |  | 0.106 |  |  | 0.493 |
| T1-T2 | 21 | 4 |  | 19 | 6 |  |
| T3-T4 | 122 | 57 |  | 124 | 55 |  |
| N stage |  |  | 0.590 |  |  | 0.909 |
| N0 | 23 | 8 |  | 22 | 9 |  |
| N＋ | 120 | 53 |  | 121 | 52 |  |
| M stage |  |  | 0.094 |  |  | 0.868 |
| M0 | 118 | 44 |  | 114 | 48 |  |
| M1 | 25 | 17 |  | 29 | 13 |  |
| Therapeutic model |  |  |  |  |  |  |
| CCRT-C | 40 | 10 | 0.968 |  |  | 0.514 |
| I-CCRT | 10 | 9 |  | 34 | 16 |  |
| I-CCRT-C | 22 | 16 |  | 12 | 7 |  |
| SCRT | 71 | 26 |  | 28 | 10 |  |
| Radiotherapy technology |  |  | 0.941 | 69 | 28 | 0.227 |
| 3D-CRT | 50 | 21 |  | 46 | 25 |  |
| IMRT | 93 | 40 |  | 97 | 36 |  |
| Radiotherapy dose (Gy) |  |  | 0.838 |  |  | 0.599 |
| <60 | 49 | 20 |  | 50 | 19 |  |
| ≥60 | 94 | 41 |  | 93 | 42 |  |
| Chemotherapy plan |  |  | 0.324 |  |  | 0.991 |
| DP | 93 | 44 |  | 96 | 41 |  |
| PF | 50 | 17 |  | 47 | 20 |  |
| Chemotherapy cycles |  |  | 0.552 |  |  | 0.782 |
| 4-5 | 93 | 37 |  | 92 | 38 |  |
| 6-8 | 50 | 24 |  | 51 | 23 |  |
| **The hematology test results** | | | | | | |
| CEA (ng/ml) |  |  | 0.071 |  |  | 0.302 |
| <3.4 | 111 | 54 |  | 108 | 46 |  |
| ≥3.4 | 32 | 7 |  | 35 | 15 |  |
| Cyfra21(ng/ml) |  |  | 0.710 |  |  | 0.986 |
| <3.3 | 109 | 45 |  | 113 | 52 |  |
| >3.3 | 34 | 16 |  | 30 | 9 |  |
| Anemia (mmol/L) |  |  | 0.126 |  |  | 0.514 |
| ≥4.9 | 143 | 60 |  | 142 | 61 |  |
| <4.9 | 0 | 1 |  | 1 | 0 |  |
| Leukopenia(*10^9^/L) |  |  | 0.066 |  |  | 0.414 |
| ≥2.0 | 94 | 48 |  | 102 | 40 |  |
| ＜2.0 | 49 | 13 |  | 41 | 21 |  |
| Thrombocytopenia（*10^9^/L) |  |  | 0.303 |  |  | 0.703 |
| ≥50 | 133 | 59 |  | 134 | 58 |  |
| ＜50 | 10 | 2 |  | 9 | 3 |  |
| Neutropenia（*10^9^/L) |  |  |  |  |  | 0.7819 |
| ≥1.0 | 86 | 44 |  | 92 | 38 |  |
| ＜1.0 | 57 | 17 |  | 51 | 23 |  |
| AST (U/L) |  |  | NA |  |  | NA |
| ＜5*ULN | 143 | 61 |  | 143 | 61 |  |
| ≥5*ULN | 0 | 0 |  | 0 | 0 |  |
| ALT (U/L) |  |  | 0.256 |  |  | 0.256 |
| ＜5*ULN | 140 | 61 |  | 140 | 61 |  |
| ≥5*ULN | 3 | 0 |  | 3 | 0 |  |
| TBIL (U/L) |  |  | 0.140 |  |  | 0.140 |
| ＜3*ULN | 138 | 61 |  | 138 | 61 |  |
| ≥3*ULN | 5 | 0 |  | 5 | 0 |  |
| **The side effects after treatment** | | | | | | |
| RE (grades) |  |  | 0.290 |  |  | 0.613 |
| 1,2 | 133 | 54 |  | 132 | 55 |  |
| 3,4 | 10 | 7 |  | 11 | 6 |  |
| RP (grades) |  |  | 0.580 |  |  | 0.487 |
| 1,2 | 133 | 58 |  | 135 | 56 |  |
| 3,4 | 10 | 3 |  | 8 | 5 |  |
| NV (grades) |  |  | 0.853 |  |  | 0.474 |
| 1,2 | 139 | 59 |  | 138 | 60 |  |
| 3,4 | 4 | 2 |  | 5 | 1 |  |
| Cardiac disorders |  |  | 0.776 |  |  | 0.485 |
| Yes | 127 | 55 |  | 129 | 53 |  |
| No | 16 | 6 |  | 14 | 8 |  |
| **Treatment response** | | | | | | |
| ORR |  |  | 0.092 |  |  | 0.007 |
| Yes | 108 | 39 |  | 111 | 36 |  |
| No | 35 | 22 |  | 32 | 25 |  |
| DCR |  |  | 0.082 |  |  | 0.023 |
| Yes | 133 | 52 |  | 134 | 51 |  |
| No | 10 | 9 |  | 9 | 10 |  |
| Response |  |  | 0.370 |  |  | 0.008 |
| CR | 9 | 8 |  | 13 | 4 |  |
| PR | 99 | 31 |  | 98 | 32 |  |
| SD | 25 | 13 |  | 23 | 15 |  |
| PD | 10 | 9 |  | 9 | 10 |  |
| Median survival time (days) | 720 (123-2886) | 576 (150-2424) | 0.418 | 1029(153-2868) | 816 (147-2886) | 0.097 |

PFS: progress free survival; OS: overall survival; ECOG PS: Eastern Cooperative Oncology Group performance status; 3D-CRT: 3-dimensional conformal radiation therapy; IMRT: intensity-modulated radiation therapy; DP: cisplatin plus docetaxel; PF: cisplatin plus fluorouracil; CEA: carcinoembryonic antigen; AST: aspartate aminotransferase ALT: Alanine aminotransferase; ULN: upper limit of normal value; TBIL: total bilirubin; RE: radiation esophagitis; RP: radiation Phe; NV: nausea/vomiting; ORR: objective response rate; DCR: disease control rate; CR: complete response; PR: partial response; NR: no response; PD: progressive disease.
