# Supplementary material for: Machine learning models predict overall survival and progression free survival of non-surgical esophageal cancer patients with chemoradiotherapy based on CT image radiomics signatures
Source: Radiat Oncol. 2022 Dec 27;17:212. doi: 10.1186/s13014-022-02186-0 (PMC9795769; doi:10.1186/s13014-022-02186-0)
Supplement: Supplementary file 6 — Additional file 6: Table S2. The clinical characteristics analysis of PFS and OS in the training cohort. [file 13014_2022_2186_MOESM6_ESM.docx]

Table S2. The clinical characteristics analysis of PFS and OS in the training cohort

|  | PFS | | | OS | | |
| --- | --- | --- | --- | --- | --- | --- |
|  | Univariate  Cox *P*-values | Multivariate  Cox *P*-values | HR | Univariate  Cox *P*-values | Multivariate  Cox *P*-values | HR |
| **Patients’ characteristic** | | | | | | |
| Age | 0.776 |  |  | 0.232 |  |  |
| Gender | 0.454 |  |  | 0.791 |  |  |
| ECOG PS | *P*<0.01 | *P*<0.01 | 2.617 | 0.017 | 0.132 |  |
| Tumor location | 0.196 |  |  | 0.53 |  |  |
| Differentiation | *P*<0.01 | *P*<0.01 | 2.287 | *P*<0.01 | 0.026 | 2.066 |
| T stage | 0.314 |  |  | 0.518 |  |  |
| N stage | 0.047 | *P*<0.01 | 1.344 | 0.023 | 0.014 | 4.284 |
| M stage | 0.024 | 0.311 |  | *P*<0.01 | *P*<0.01 | 3.376 |
| Radiotherapy technology | 0.332 |  |  | 0.247 |  |  |
| Radiotherapy dose | 0.113 |  |  | 0.306 |  |  |
| Chemotherapy plan | 0.958 |  |  | 0.160 |  |  |
| Chemotherapy cycles | 0.283 |  |  | 0.572 |  |  |
| Therapeutic model | 0.019 | 0.101 |  | 0.198 |  |  |
| **Hematology test results parameters** | | | | | | |
| CEA | 0.780 |  |  | 0.511 |  |  |
| Cyfra21 | 0.011 | 0.915 |  | 0.049 |  |  |
| Anemia | NA |  |  | 0.579 |  |  |
| Leukopenia | 0.473 |  |  | 0.498 |  |  |
| Thrombocytopenia | 0.934 |  |  | 0.371 |  |  |
| Neutropenia | 0.546 |  |  | 0.128 |  |  |
| AST | NA |  |  | NA |  |  |
| ALT | 0.407 |  |  | 0.996 |  |  |
| Total bilirubin | 0.507 |  |  | 0.995 |  |  |
| **The side effects after treatment** | | | | | | |
| RE | 0.035 | *P*<0.01 | 4.617 | 0.036 | 0.181 |  |
| RP | 0.070 | 0.870 |  | 0.034 | 0.362 |  |
| NV | 0.516 |  |  | 0.791 |  |  |
| Cardiac disorders | 0.240 |  |  | 0.927 |  |  |
| **The treatment response** | | | | | | |
| ORR | *P*<0.01 | *P*<0.01 | <0.001 | *P*<0.01 | 0.733 |  |
| DCR | *P*<0.01 | *P*<0.01 | <0.001 | *P*<0.01 | 0.700 |  |
| Response | *P*<0.01 | *P*<0.01 | >1000 | *P*<0.01 | 0.129 |  |

PFS: progress free survival; OS: overall survival; ECOG PS: Eastern Cooperative Oncology Group performance status; DP: cisplatin plus docetaxel; PF: cisplatin plus fluorouracil; CEA: carcinoembryonic antigen; AST: aspartate aminotransferase ALT: Alanine aminotransferase; RE: radiation esophagitis; RP: radiation [pneumonitis](http://www.baidu.com/link?url=Utm5xiE75oykB94GWqrwh8I3adGtz16Z5H0_qTakK9ismWGO9ifGvqdrQY_9DMvcn7X5b2do2quNp_8MRd99gHBxKHdXTwuIcZ5Xfvt-Ec1KNEe7YTr3C3xKEevojvRk); NV: nausea/vomiting; ORR: objective response rate; DCR: disease control rate;
