# Supplementary material for: Machine learning models predict overall survival and progression free survival of non-surgical esophageal cancer patients with chemoradiotherapy based on CT image radiomics signatures
Source: Radiat Oncol. 2022 Dec 27;17:212. doi: 10.1186/s13014-022-02186-0 (PMC9795769; doi:10.1186/s13014-022-02186-0)
Supplement: Supplementary file 7 — Additional file 7: Table S3. Radiomics features selected with LASSO Cox for PFS prediction. [file 13014_2022_2186_MOESM7_ESM.docx]

Table S3. Radiomics features selected with LASSO Cox for PFS prediction

| Radiomics feature name | Training cohort | Test cohort | *P*-values |
| --- | --- | --- | --- |
| OSM | 141.22(26.833~340.801) | 139.460(34.986~298.918) | 0.984 |
| WHGG | 152.695(9.200~1439.634) | 169.416(12.364~841.310) | 0.820 |
| WHGL | -0.070(-0.114~-0.020) | -0.070(-0.104~-0.032) | 0.604 |
| WGD | 0.434(-0.091~0.619) | 0.445(0.373~0.596) | 0.5864 |
| WLGG | 341.425(23.899~4285.074) | 384.339(21.728~2825.914) | 0.656 |
| WLGL | 31.695(3.319~3610.704) | 22.363(4.356~3912.983) | 0.846 |

LASSO: least absolute shrinkage and selection operator; OSM: original, shape, Maximum2DDiameterRow; WHGG: wavelet-HLH, glszm, GrayLevelNonUniformity; WHGL: wavelet-HHL, glcm, Imc1; WGD: wavelet-HHH, gldm, DependenceNonUniformity; WLGG: wavelet-LLL，gldm，GrayLevelNonUniformity; WLGL: wavelet-LLL,glszm, LargeAreaEmphasis;
