# Supplementary material for: Machine learning models predict overall survival and progression free survival of non-surgical esophageal cancer patients with chemoradiotherapy based on CT image radiomics signatures
Source: Radiat Oncol. 2022 Dec 27;17:212. doi: 10.1186/s13014-022-02186-0 (PMC9795769; doi:10.1186/s13014-022-02186-0)
Supplement: Supplementary file 8 — Additional file 8: Table S4. Radiomics features selected with LASSO Cox for OS prediction. [file 13014_2022_2186_MOESM8_ESM.docx]

Table S4. Radiomics features selected with LASSO Cox for OS prediction

| Radiomics feature name | Training cohort | Test cohort | *P*-values |
| --- | --- | --- | --- |
| OSM | 141.223(26.833~340.801) | 146.632(54.332~277.042) | 0.828 |
| WHGG | 152.695(9.200~1439.634) | 140.012(22.340~789.143) | 0.976 |
| WLGG | 341.425(23.899~4285.074) | 329.386(33.497~3058.499) | 0.5927 |

LASSO: least absolute shrinkage and selection operator; OSM: original, shape, Maximum2DDiameterRow; WHGG: wavelet-HLH, glszm, GrayLevelNonUniformity; WLGG: wavelet-LLL，gldm，GrayLevelNonUniformity
